# Modern Problems in Psychiatry

**Published:** 1910-06

**Authors:** 


**Modern Problems in Psychiatry.** By ERNESTO LUGARO. Translated by DAVID ORR, M.A., and R. G. ROWS, M.D. With a Foreword by T. S. CLOUSTON, M.D. Pp. vii, 305. Manchester: at the University Press. 1909.

A merely perfunctory notice of the translators' task is in this case insucffiient. Drs. Orr and Rows deserve full recognition of their masterly rendering into English. The language of the author is so concise, absolute precision is so vital in expressing intelligibly his subtle and involved reasoning, the intimate knowledge of the subject treated is so essential, that exceptional abilities have been required in the work of translation. This production of Lugaro's is in every way remarkable and admirable. In a volume of very moderate dimensions he has reviewed most of the realm of psychiatry and placed under special survey the problems chiefly deserving attention. A preface by Dr. Clouston forms a commentary and epitome of a work which is almost beyond reviewing; our purpose here can be no more than to notice its main features and tendencies.

Prominent is Lugaro's materialism, the inevitable result to his sanguine mind of the steady aggregation of physico-pathological findings in the nervous system. To him the somatic side of insanity only awaits revelation, and whilst appreciative of the intense difficulties of the task, he is hopefully sustained by discoveries already made, and is fruitful of hypotheses and suggestions for future investigators. No feeling of mysticism daunts, no impassable barrier between matter and mind presents itself to him. The whole tendency of his work is to inculcate a habit of regarding symptoms and their physical correlatives in parallel, and to estimate the disease in terms of the latter.

Lugaro's full comprehension of current literature enables him to condense into lucidly-expressed but brief space all that is worthiest in recent research. The structure and functions of the neurones are clearly discussed; their relationship to the external world; their intra-neuronic modifications, or affective tone, in regard to organic states ; the nature and importance of the neuroglia ; the hypotheses of dynamic polarisation, avalanche, chemiotropism, associationism, reciprocal currents between related centres, and many other subjects are treated of. A careful consideration is given to the determination of the nature and sites of morbid lesions, and to the needful caution to be exercised in estimating the influences of disturbances provoked secondarily or by a common cause, such as toxæmia. Thus in senile dementia and general paralysis the involvement of nerve-elements is unsystematic and diffuse, and the symptoms are complex, both by reason of this irregularity and of the nutritional implication. That even the simplest images have an extensive representation in the cortex has also to be remembered. Again, lesions more limited in zone or system are indicated in dementia precox (probably a primary disease of cortical neurones), or in systematised delusions. Lugaro lays stress on the dynamic or functional disturbances underlying affective states, the primary spontaneous character of the latter, and their paramount influence on ideation. Congenital or acquired, they exercise their dynamics in coercing all images and associations to harmony with their prevalent tone ; a biased view is thus presented to consciousness.

The author places the origin of fixed ideas in the apprehensive affective state aroused by their persistence. His theory of hallucinations is that they are dependent on over-functionating in the re-presentative centres which register those images formed in the perceptive centres ; that is, an over-energising of the stimuli reflected from the former arouses images in the latter, similar to those produced by external objects.

The section on etiology is as encouraging as valuable. All endogenous and exogenous causes of mental disease are reviewed, and Lugaro evolves a lengthy list of influences—material, assailable or eliminable—offering a hopeful prospect for treatment, particularly of a prophylactic kind. Lugaro's views on inheritance are indicated in his statements that heredity tends to perpetuate unaltered the products of biological experience, and the adaptations that have been acquired during the evolution of life. Degeneration, then, is a disease of the stock, acquired through external influences, and may be recovered from by the inverse process of regeneration. One would assume, however, that this opinion must be accepted conditionally as being a matter of degree. The author then favours the hereditary transmission of acquired qualitative somatic characters, and the transformation of functional hypertrophies into normal stabile growths, a correlative influence, continuous and dynamic, existing between every organ and tissue and germ-plasm. The acquisitions are probably strengthened and fixed through the operation of natural selection, whilst it is owing to the establishment of a physiological equilibrium that the progressive transmission of functional hypertrophies becomes arrested.

A strong and hopeful plea for the corrective influence of a healthy environment is advanced. Recognising the merely provisional terminology of many of the so-called "syndromes," Lugaro admits but a small list of clinical entities; it is the conception of the disease itself that he seeks to establish, the study of the cerebral and extra-cerebral organic processes underlying that is of importance.

His final chapter on practical problems is a general disquisition on the main principles of prophylaxis and treatment, on asylum administration, the relations between insanity, crime, punitive and deterrent measures, and so forth, together with some stringent criticisms of the attitude of expert witnesses in medico-legal cases. It is impossible to do justice in a brief notice to the merits of this book. It has long been awaited, and will doubtless meet with every success. The publishers have done their part admirably.

